# Thermal Desorption–Vocus Enables Online Nondestructive Quantification of 2,4,6-Trichloroanisole in Cork Stoppers below the Perception Threshold

**DOI:** 10.1021/acs.analchem.0c01326

**Published:** 2020-06-10

**Authors:** Luca Cappellin, Felipe D. Lopez-Hilfiker, Veronika Pospisilova, Luigi Ciotti, Paolo Pastore, Marc Gonin, Manuel A. Hutterli

**Affiliations:** † Dipartimento di Scienze Chimiche, Università degli Studi di Padova, via Marzolo 1, 35131 Padova, Italy; ‡ Research and Innovation Centre, Fondazione Edmund Mach, via Mach 1, 38010 San Michele All’adige, Italy; § Tofwerk AG, Schorenstrasse 39, CH-3645 Thun, Switzerland

## Abstract

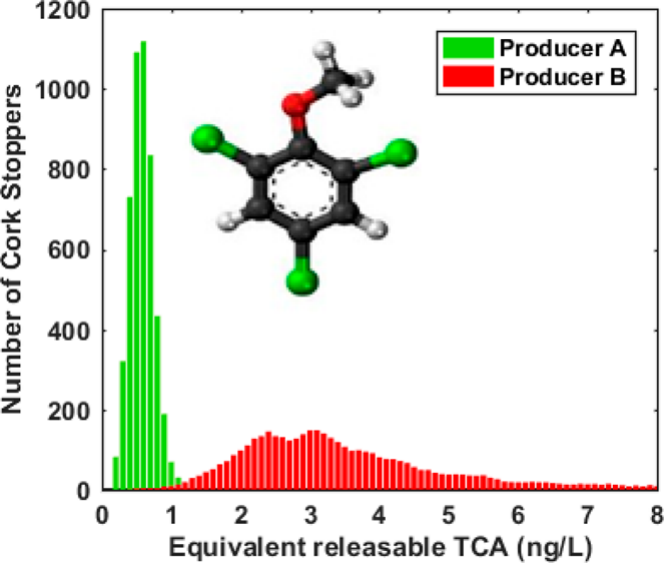

2,4,6-Trichloroanisole (TCA) contamination of wine determines huge economic losses for the wine industry estimated to amount to several billion dollars yearly. Over 50 years of studies have determined that this problem is often caused by TCA contamination of the cork stopper, which releases TCA into the wine. The human threshold for TCA is extremely low. A wine contaminated by 1–2 ng/L TCA can be perceived as tainted. Contaminations with <0.5 ng/L TCA are commonly considered negligible and are not perceivable. The possibility of prescreening cork stoppers for TCA contamination would be an enormous advantage. Therefore, the demand for a fast, nondestructive method capable of quantifying the TCA contamination in cork stoppers is impelling. Vastly used analytical methods have so far struggled to provide a fast and reliable solution, whereas sensory analysis by trained panelists is expensive and time-consuming. Here we propose a novel approach based on chemical ionization–time-of-flight (CI-TOF) mass spectrometry employing the “Vocus” ion source and ion–molecule reactor. The technique proved capable of nondestructively quantifying TCA contamination in a single cork stopper in 3 s, with a limit of quantification below the perception threshold. A real test on the industrial scale, quantifying TCA contamination in more than 10000 cork stoppers in a few hours is presented, representing the largest data set of TCA analysis on cork stoppers within the literature and proving the possibility to apply the technique in an industrial environment. The correlation with standard methods for releasable TCA quantification is also discussed.

Cork taint in wine mainly caused by 2,4,6-trichloroanisole (TCA) is a tremendous problem for the cork and wine industry causing annual losses exceeding 10 billion dollars.^1^ Despite wine being a complex matrix constituted by hundreds of different aroma compounds, the presence of a few ng/L of TCA may completely spoil the wine.^2^ The discovery of TCA as a compound responsible for cork taint in wine dates back to the early 1980s.^3^ Since then, it has been established that TCA is responsible for the majority of the wines spoiled by cork taint, but other compounds can also be possible causes. For example, Chatonnet et al.^4^ identified 2,4,6,-tribromoanisole (TBA) as causing musty or corked taint in wine that did not contain significant levels of chloroanisoles, confirming suggestions from earlier studies.^5^ 2,4-dichloroanisole (2,4-DCA), 2,6-dichloroanisole (2,6-DCA), 2,3,4,6-tetrachloroanisole (TeCA), and pentachloroanisole (PCA) in the cork might also taint the wine but their role is mostly minor.^2^

Despite almost 30 years of investigations,^6−10^ the ultimate reasons for the presence of TCA in wine are not fully understood.^11^ To date, only TCA transferred from the cork stopper into the wine has been identified, as experiments trying to demonstrate TCA formation from precursors (trichlorophenols) in wine have shown that it is not the case.^12^ The presence of TCA and other chloroanisoles in the corkwood is still largely unknown as well as responsible microorganisms and the timing of the formation.^11^ Precursors may originate from chlorophenolic biocides and be transformed into chloroanisoles by several microorganisms.^13^ Moreover, they may be formed from compounds naturally occurring in wood or cork through chlorination and microbial methylation reactions.^13^ It is common knowledge among cork producers that the incidence of TCA is higher in unmanaged cork oak forests with a strong presence of low vegetation than in managed forests subjected to regular cleaning operations. Cork stoppers produced from the same bark may be contaminated with highly variable levels of TCA since its presence is strongly localized.^13^ Therefore, the level of contamination must be determined in single cork stoppers, as TCA screening of the bark would either result in unnecessary cork discards or would often overlook localized TCA contaminations.

Prescott et al.^14^ determined the consumer rejection threshold and sensory detection threshold for TCA taint in wine to be 3.1 and 2.1 ng/L, respectively. Later studies suggested that the sensory detection threshold could be even lower, around 1 ng/L.^15^ Therefore, TCA screening of single cork stoppers at an industrial scale poses a serious analytical challenge. A fast, sensitive, accurate, nondestructive analytical method is required but at present, despite many attempts, a break-through in cork screening at an industrial scale has not been realized. Since only a small part of the TCA content of the cork stopper is able to migrate to the wine,^16−18^ a common approach adopted by the cork and wine industries is the analysis of releasable TCA.^2^ Standards of releasable TCA analysis are described by ISO 20752:2014 and by the OIV organization.^20^ Basically, a cork stopper is subjected to a 24 h soaking in a wine-simulant (or, alternatively in the OIV standard, in white wine) and the concentration of TCA is determined (in ng/L) in the extracting solution by either SPME-GC-MS or SPME-GC-ECD. The detection limits of such methods are typically in the range 0.2–0.5 ng/L^21^ and therefore are suitable for the application. In contrary, sample preparation time, analysis time, and sample destruction/modification make them unsuitable for nondestructive industrial screenings and limit their application solely to laboratory analyses (in industry quality control laboratories and external laboratories).

The methods for releasable TCA analysis in cork stoppers have been recently reviewed.^2,22,23^ Traditional methods are typically based on gas chromatography after extraction in a suitable solvent. Preconcentration is typically achieved using stir bar sorptive extraction,^24^ solid phase extraction,^25^ or, more often, solid phase microextraction (SPME).^26^ Detection is then performed by Electron Capture Detector (ECD)^27^ or by mass spectrometry.^26,28^ Evolutions of the technique introduced microwave assisted extraction over 2 h in place of the 24 h soaking, thus, strongly decreasing the required sample preparation time.^24^ Biosensors have also been proposed, employing specific TCA antibodies to analyze TCA in about 5 min. They could detect TCA in cork soaks at concentrations as low as 1.02 ng/L.^29,30^ Since releasable TCA is considered a destructive technique leading to surface modifications,^15^ the industry is aiming for methods that are nondestructive, fast, and correlate with releasable TCA. The most promising approaches in this direction involve the analysis of gaseous TCA released by a single cork stopper in a vial or other closed container upon heating.^31−33^ Strong technological investments by major cork producers in direct GC analysis provided solutions claiming to screen single cork stoppers in 20 s at releasable TCA levels not exceeding 0.5 ng/L^2^ but are still leading to controversial outcomes (e.g., Todorov and Courtwatch^34^). Some studies introduced chromatography-free techniques to tackle the problem. The potential of spectrometric techniques based on ion mobility in this field has been first proposed^35^ and then subsequently further studied.^36^

A large role in nondestructive industrial TCA screenings is played by sensory analysis. Different approaches exist. The standard method^37^ implies partial soaking in distilled water for 24–48 h, followed by sensory analysis. According to the so-called dry soak approach, single cork stoppers are placed in a closed vial with some drops of water for 24 h, and the headspace air is subsequently tested by trained panelists.^15^ Other common practices within the industry include preheating of dry corks at 150 °C for 10 min before sensory analysis, preheating of maceration vessels at 80 °C for 2 h, or the use of sparkling water instead of distilled water for maceration. However, sensory approaches are time-consuming and expensive.^2^ Moreover, the “sensory approaches” used today in the cork industry are rather “sniff tests” focused on high capacity production (typically 2000 corks/day/panelist), using a single trained panelist per single sample, and are therefore subjective, lacking any of the criterium of objectivity. Further, such approaches unavoidably lead to sensory saturation and, therefore, to false negatives and positives, as TCA is an olfactive suppressor agent.^38^ In order to obtain objective results, the quantitative descriptive analysis or flavor profile of any food or food-related single sample should be estimated by means of a sensory team composed of 8–12 trained panelists,^39^ and the repeatability and the reproducibility of the sensory panel should be regularly and continuously tested by the panel leader according to standard guidelines.^40^ Objective sensory sessions are thus slow and have a very low capacity.

The present study is the first exploitation of a novel method for the online detection of TCA based on chemical ionization mass spectrometry (CI-MS). This method omits the use of chromatography and recovers the separation by high resolution mass spectrometry and by selective and soft ionization. The CI source in CI-MS involves the production of reagent ions in a dedicated reagent ion source. The reagent ions are then transferred into an ion–molecule reactor (IMR) where they encounter the analyte and produce the analyte ions by chemical ionization. The instrument used here uses a discharge reagent ion source and an IMR operated at a medium pressure of ≈1.5 mbar^41^ coupled to time-of-flight mass spectrometry (TOFMS). Krechmer et al.^42^ recently described the performance and the potential of the new instrument (Vocus 2R, from Tofwerk, Switzerland) when used with proton transfer reaction chemical ionization, showing a more than 10-fold sensitivity improvement for volatile compound detection compared to current state-of-the art instruments. Here, chemical ionization via electron transfer to NO^+^ reagent ions is employed to ionize TCA. The sampling from gaseous cork emission is done without preconcentration or trapping. The method, therefore, strongly resembles the sensory analysis mentioned above, but using an artificial rather than a human nose.

The aim of the study is 3-fold. We first show that the Vocus can detect TCA in natural cork stoppers at concentrations below the sensory threshold in 3 s. The correlation with releasable TCA determinations according to ISO 20752:2014^19^ and OIV^20^ is then investigated. Finally, a real industrial scenario is simulated by determining TCA in 10100 natural cork stoppers from three different batches in just 8 h and 25 min, corresponding to 3 s per cork stopper.

## Materials and Methods

### Cork Stoppers

Natural cork stopper samples (24 mm diameter, 49 mm length) were obtained directly from several producers. Cork visual grades ranged from Flor to II according to the international guide.^43^ All stoppers were not coated; therefore, no material other than cork was present. Calibrations, method testing, and comparison with other TCA analysis methods were performed on natural cork stoppers from a mixed batch. The testing in an industrial scenario was performed on a mixed batch (100 natural cork stoppers) from different producers and on two homogeneous batches each consisting of 5000 natural cork stoppers from the same producer.

### Vocus Cork Analyzer (VCA)

The VCA (Tofwerk AG, Switzerland) includes a Vocus 2R high resolution chemical ionization mass spectrometer coupled to a cork autosampler. The Vocus 2R reaches a mass resolving power of up to m/dm = 15000. The discharge reagent-ion source was operated at ≈2 mbar and generated NO^+^ reagent ions from synthetic air (Alphagaz 1 Air, Air Liquide). The IMR was operated at 1.5 mbar and 150 °C. The ion transmission into the TOFMS was optimized using radio frequency ion focusing.^42,44,45^ TCA ions are produced with chemical ionization (CI) via charge transfer. Fragmentation of the analyte ions was negligible at the selected setup. The cork stopper autosampler consists of individual cork stopper cavities to prevent cross contaminations. The cavity temperature was generally set to 120 °C, unless otherwise stated. The Vocus Cork Analyzer was set to measure for 2 s from each cavity drawing headspace air at 1 slpm (= standard liters per minute) through a PTFE sampling line (1/8 in. i.d.) heated at 120 °C.

Simultaneously, synthetic air was flushed into the cavity in order to replace the sampled headspace gas. The settling time between cavities was 1 s, resulting in a total cycle period of Δ*t* = 3 s/cork stopper. A total of 5 sccm (sccm = standard cubic centimeters per minute) of 10 μL/L (ppmv = parts per million by volume) benzene, toluene, and xylene in pure nitrogen from a standard cylinder (Carbagas, Switzerland) was mixed into the sample flow in order to monitor the primary ion stability. The signal intensity expressed in counts per second (cps) of the spectral peaks at 209.940 Th (corresponding to C_7_H_5_Cl_3_O^+^, 1 Th = 1 Da/e), 211.937 Th (isotope of C_7_H_5_Cl_3_O^+^), and 213.934 Th (isotope of C_7_H_5_Cl_3_O^+^) were summed and used as signal for TCA. The benzene signal C_6_H_6_^+^ was used as an internal standard in order to correct for possible sensitivity drifts. The conversion into equivalent releasable TCA expressed in ng/L was carried out upon calibration against the standard method (see next sections).

### Releasable TCA Analysis According to Standard Methods

Releasable TCA in natural cork stoppers was determined as described in OIV-MA-AS315-16.^20^ The used methodology is also in accordance to ISO 20752:2014^19^ with a minor modification; that is, the only deviation from the ISO 20752:2014^19^ prescriptions was the fact that we avoided rounding the results to the “nearest 0.5”, which is actually not in the provisions of the OIV method. The procedure was as follows. A single cork stopper was placed into a 50 mL aqueous-alcoholic solution (12% v/v alcoholic strength) for 24 h. In the case of cork granules, 40 g of granules were placed in a 2 L flask and completely covered with the aqueous-alcoholic solution, soaking for 24 h. A total of 10 mL of solution was inserted into a 20 mL vial, adding 3 g of NaCl and 100 mL of internal standard solution consisting of 10 ng/L 2,4,6-trichloroanisole-*d*_5_ (TCA-d5) in an aqueous-alcoholic solution, 12% v/v. The vial was kept under stirring at 35 °C. Headspace volatile compounds were collected for 15 min by a 2 cm Solid Phase Microextration fiber coated with divinylbenzene/carboxen/polydimethylsiloxane 50/30 μm (DBV/CAR/PDMS, Sigma-Adrich, St. Louis, U.S.A.). Volatile compounds adsorbed on the SPME fiber were desorbed at 260 °C for 2 min in splitless mode in the injector port of a GC interfaced with a mass detector (GC Agilent 7820A with Agilent 5977B MSD, Agilent Technologies, Santa Clara CA, U.S.A.). Separation was achieved on an Agilent HP-5 capillary column (30 m × 0.25 mm ID × 0.25 μm film thickness; Sigma-Adrich, St. Louis, U.S.A.).

The GC oven temperature program consisted of 35 °C for 6 min, then 35–280 °C at 15 °C min^–1^, and stable at 280 °C for 5 min. Helium was used as the carrier gas with a constant column flow rate of 1 mL min^–1^. The mass detector was operated in electron ionization mode (EI, internal ionization source; 70 eV) in single ion mode. 195 Th, 210 Th, and 212 Th were used to detect TCA and 199 Th, 215 Th, and 217 Th were used to detect TCA-d5. 195 Th and 215 Th were employed for quantification of TCA and TCA-d5, respectively. All reagents were purchased from Sigma-Aldrich (St. Louis, U.S.A.). The used method is widely employed within the cork industry and by accredited laboratories offering releasable TCA analysis in cork stoppers. It is, therefore, of utmost importance within the cork and wine sectors, as it is also employed (also in court) to demonstrate that the stopper of a TCA-tainted wine bottle is contaminated by TCA. In the following, the used method will be generally referred to as “ISO”.

## Results and Discussion

### TCA Determination in Natural Cork Stoppers with Vocus

**Figure 1:**
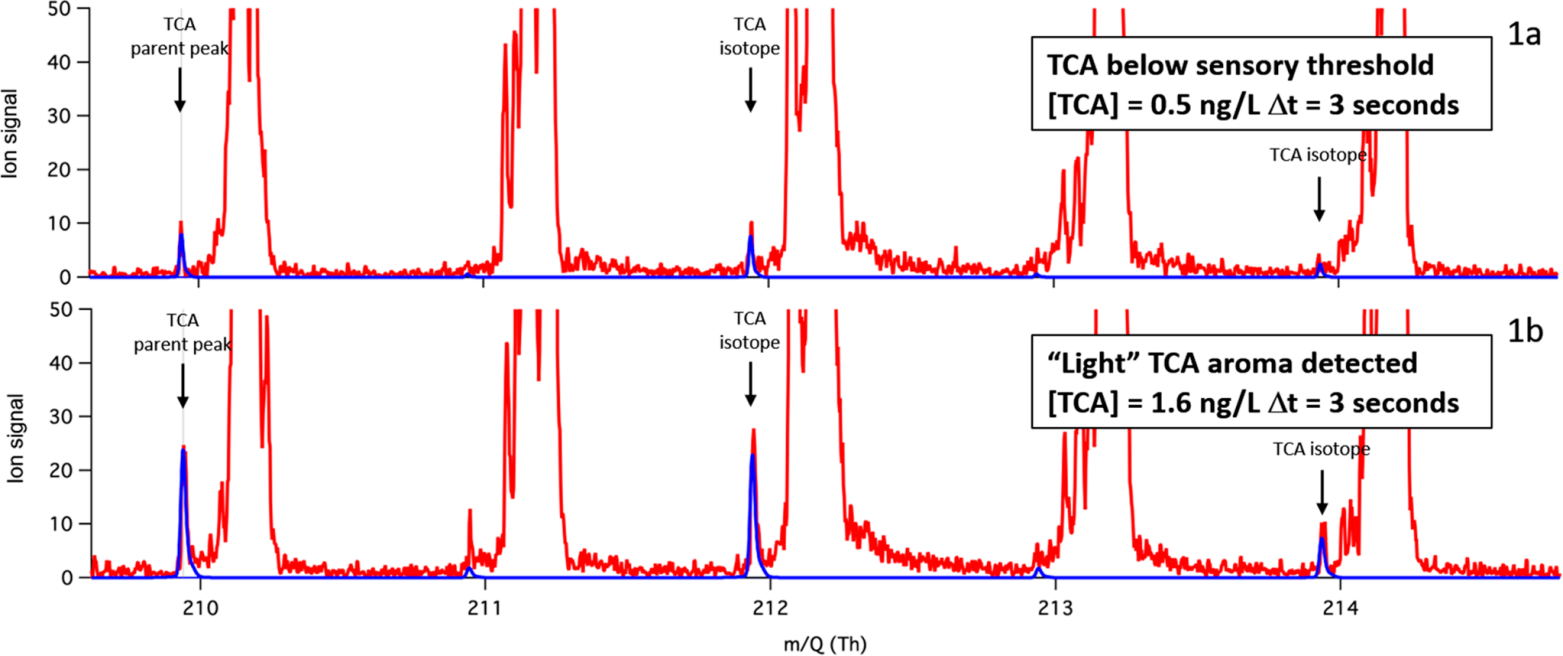
Excerpt of Vocus-measured mass spectra (red) of two natural cork stoppers. The upper spectrum corresponds to a natural cork stopper determined to have 0.5 ng/L releasable TCA, according to OIV-MA-AS315-16.^20^ The bottom spectrum corresponds to cork stopper tainted with 1.6 ng/L of releasable TCA. In blue is the theoretical isotopic distribution of TCA for comparison.

Figure 1 reports exemplificative spectra of natural cork stoppers measured by the presented methodology. The first cork stopper (Figure 1a) was determined to have 0.5 ng/L of releasable TCA using the ISO method. This value is lower than the human sensory threshold for TCA.^14,15^ The second cork stopper (Figure 1b) was measured to have 1.6 ng/L of releasable TCA, which is close to the sensory threshold. In both cases and despite the short analysis time of 3 s, both spectra show a clear presence of TCA-related peaks at 209.940 Th (corresponding to a positively charged molecule of TCA, C_7_H_5_Cl_3_O^+^) and at 211.937 Th and 213.934 Th corresponding to the isotopes of TCA C_7_H_5_Cl_2_^37^ClO^+^ and C_7_H_5_Cl^37^Cl_2_O^+^ respectively.

**Figure 2:**
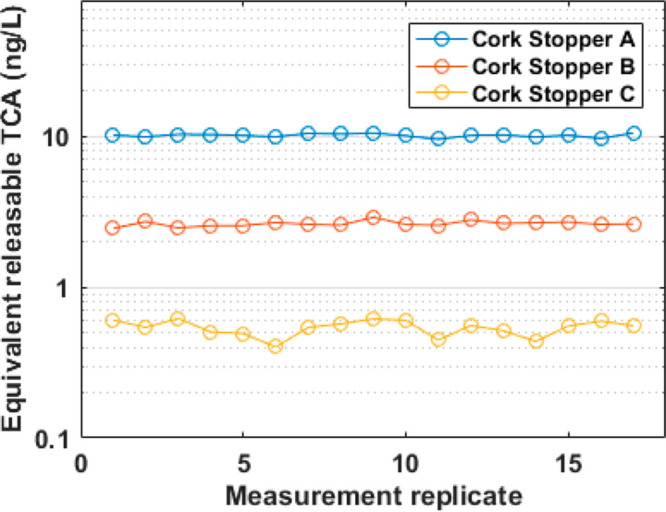
Repeatability of Vocus for TCA assessment of three natural cork stoppers having different values of releasable TCA and preheated at 120 °C.

**Figure 3:**
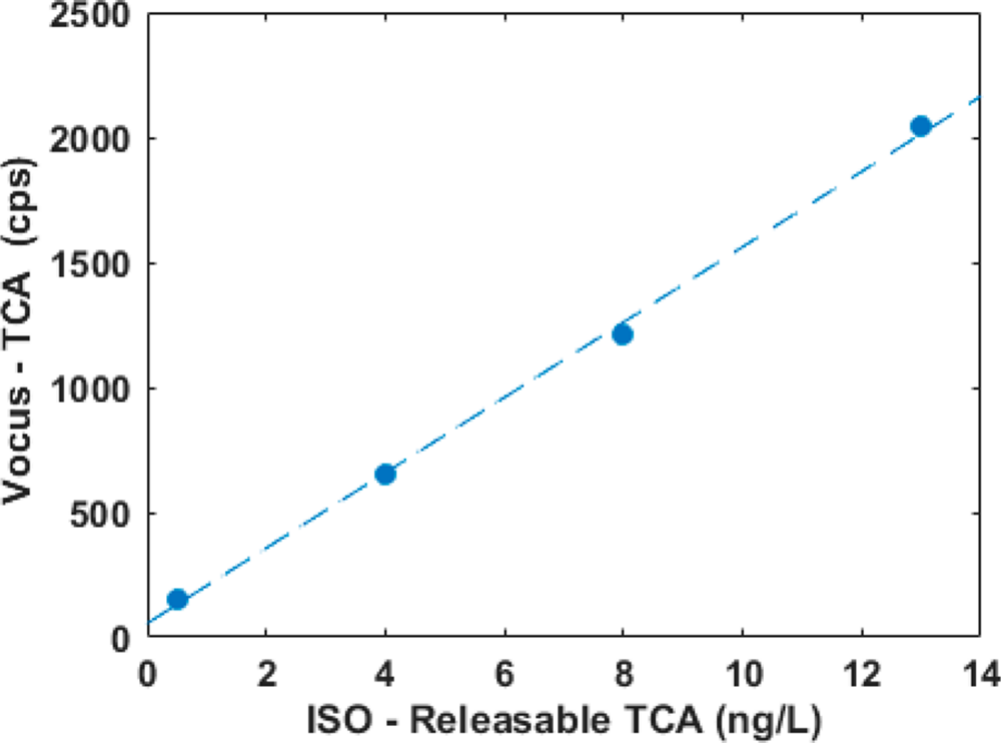
Example of linearity of the Vocus TCA signal for cork granules vs releasable TCA determined using SPME-GC-MS, as described in OIV-MA-AS315-16.^20^ The dotted line represents a linear fit (*R*^2^ = 0.998).

A Vocus spectrum of a pure TCA standard (Sigma-Adrich, St. Louis, U.S.A.) is shown in Supporting Information, Figure S1, together with the theoretical isotopic pattern showing a very good agreement. Fragmentation of TCA-related parent ions was determined to be <5%; thus, only the above-mentioned three major parent ions were considered. In principle, a limitation of the proposed method would be the fact that isomers of TCA would be superimposed on the same exact masses, and the method would measure the sum of all isomers. However, no isomers of TCA have been reported in natural cork so far, to the best of our knowledge. Some TCA isomers (for example, 2,3,6-TCA) are suggested as possible internal standards^20^ due to their absence in natural cork. Repeatability of the measurements for three natural cork stoppers preheated at 120 °C is reported in Figure 2. Relative standard deviations are 2.7%, 4.2%, and 12% for the three corks having releasable TCA of 10, 2.6, and 0.5 ng/L, respectively. Linearity of the Vocus covers six orders of magnitude, which has been demonstrated elsewhere;^42^ here, it is relevant to assess linearity of TCA determinations by Vocus within the actual matrix (natural cork) and within the relevant range for cork screening. Since the ISO methodology used to determine the amount of releasable TCA in natural cork stoppers is affected by the uncertainty in the step of soaking the cork stopper in a wine simulant solution (see next section), linearity of the present method was first assessed on cork granules (ca. 1 mm diameter) previously characterized by ISO. Figure 3 exemplifies the good linearity (*R*^2^ = 0.998) of the Vocus in the relevant range of 0.5–10 ng/L.

From the characteristics of the instrument,^42^ it can be calculated that the linearity range would extend to about 5000 ng/L of releasable TCA.

The next section reports the correlation of Vocus and ISO for releasable TCA determination on natural cork stoppers.

**Figure 4:**
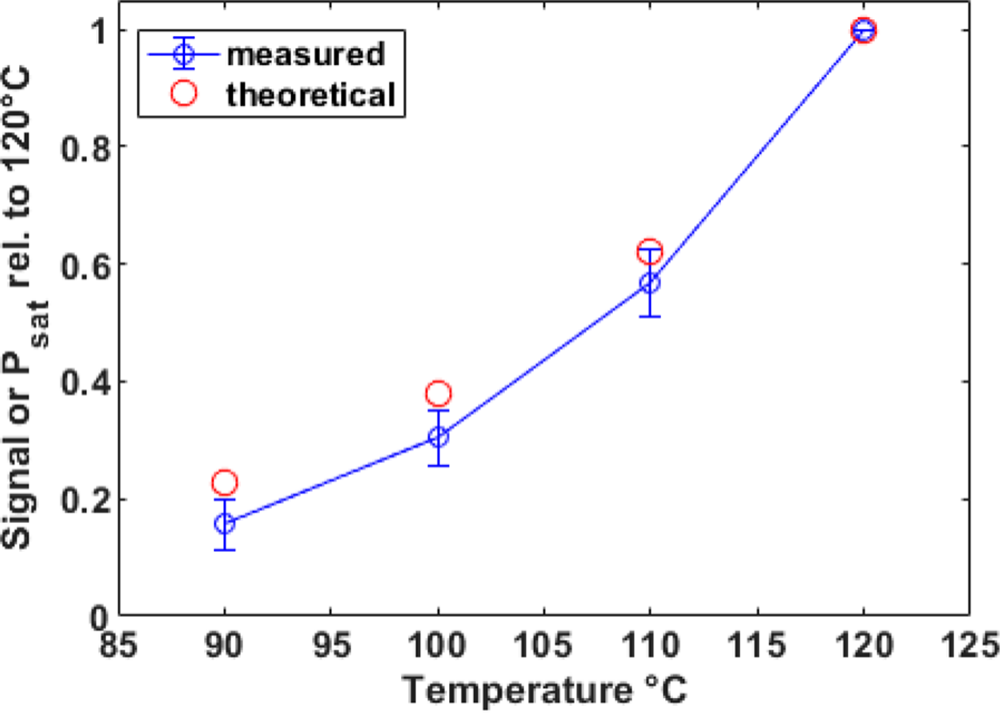
Dependence of TCA sensitivity on the natural cork stopper preheating temperature (blue). The theoretical dependence of TCA saturation vapor pressure on temperature is also reported (red). Data are normalized by the corresponding values at 120 °C.

The limit of detection was determined to be 0.05 ng/L by repeatedly (12 times) measuring 10 natural cork stoppers preheated at 120 °C for which the TCA signal was at background level and therefore considered as TCA-free samples (Table S1). The LOD was estimated as 3× the standard deviation of the repeated measurement of each cork, divided by the sensitivity. Compared to other methods available in the literature, the LOD of the present methodology is comparable or better,^2,21−23^ with the advantage being that it is achieved in just a 3 s cycle period. The sensitivity of the method depends very much on the cork stopper preheating temperature. Figure 4 reports the ratios between the sensitivities at different preheating temperatures (90, 100, and 110 °C) and the sensitivity at 120 °C. Switching the preheating temperature from 120 to 90 °C, the sensitivity drops to less than 20%. In a first approximation, the behavior of such a ratio can be explained by the change in saturation vapor pressure of TCA. The saturation vapor pressures (*P*_sat_) of TCA for selected temperatures were calculated via the Clausius–Clapeyron relationship. The enthalpy of vaporization (Δ*H*_vap_) for TCA was determined as ∼60 kJ/mol based on tabulated values of *P*_sat_ of TCA at 20 °C (3.066 Pa) and its boiling point of 241 °C.^46^

### Comparison with ISO 20752:2014^19^ and OIV^20^

**Figure 5:**
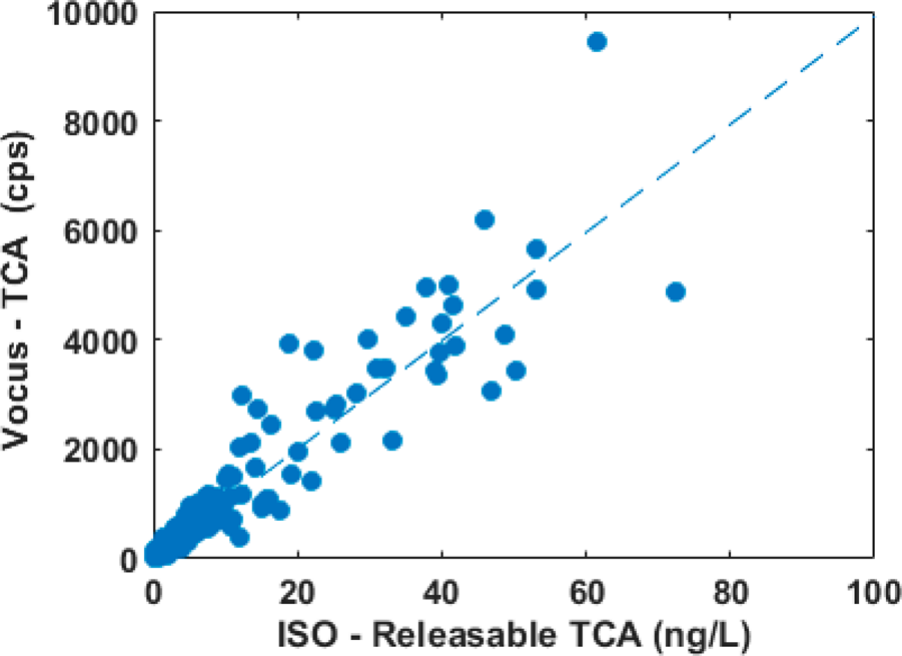
Comparison between releasable TCA determined using SPME-GC-MS, as described in OIV-MA-AS315-16,^20^ and by Vocus on a set of natural cork stoppers.

**Figure 6:**
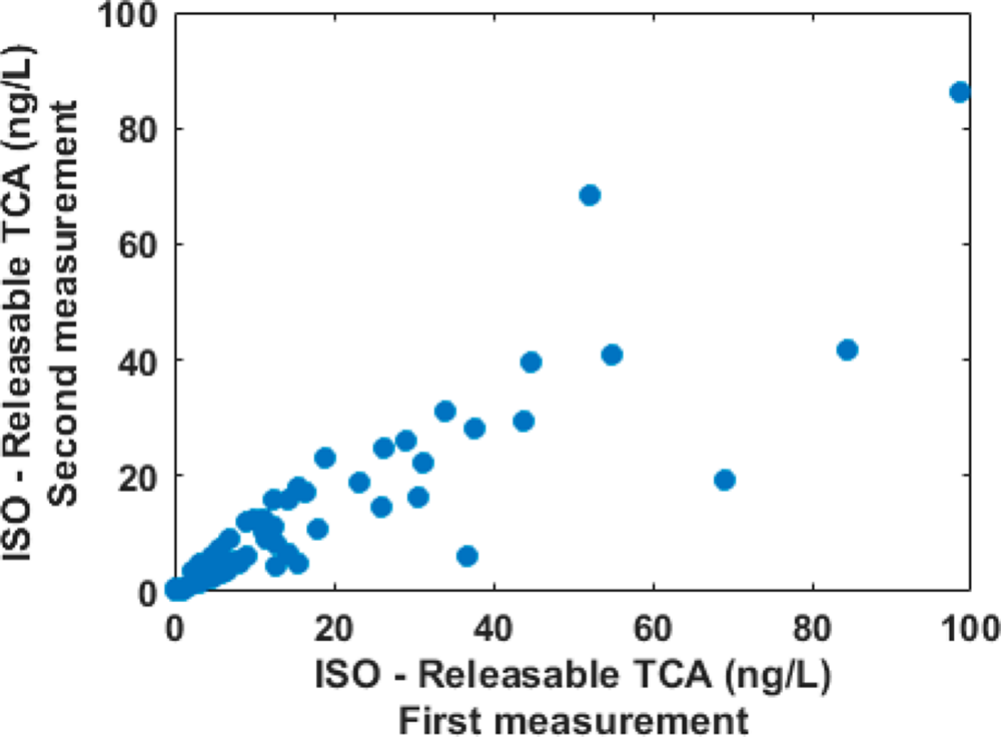
Replicate measurements of a set of 74 natural cork stoppers according to OIV-MA-AS315-16.^20^ All measurements have been carried out waiting 15–30 days before the replicated analysis.

A set of 671 natural cork stoppers was tested with the present method and by ISO. Figure 5 reports the correlation between Vocus and ISO for such data sets. *R*^2^ values of the linear fit are 0.92, implying a Pearson correlation coefficient of 0.96. The correlation is statistically significant (*p* < 0.01). In order to better evaluate such correlation results and to have a benchmark, a set of cork stoppers was measured twice according to ISO (Figure 6), waiting a period of 15–30 days between measurements. The *R*^2^ of the best fitting line is 0.68, and the Pearson correlation is thus 0.82 (*p* < 0.01). Such values suggest that the correlation between Vocus and ISO is limited by the precision of ISO. Considering the results obtained with cork granules (see Figure 3) and the good precision of ISO when measuring standard TCA solutions or cork soaks,^21^ the uncertainty is likely derived from the sample preparation step, that is, the soaking step. In such step each natural cork stopper is subjected to soaking for 24 ± 2 h in a wine simulant solution. By eliminating the soaking step, Vocus has the advantage of reducing the uncertainty related to this step.

### Application to an Industrial Scenario

**Figure 7:**
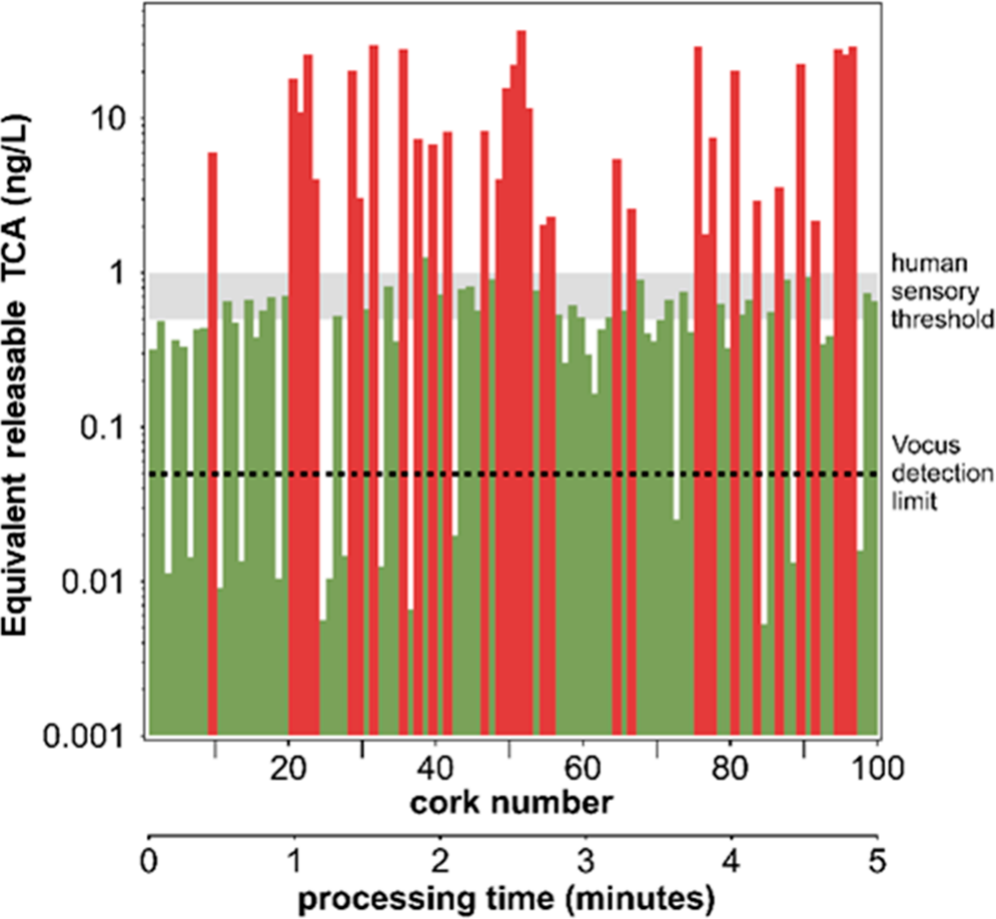
Example of an analysis of 100 natural cork stoppers measured by Vocus. The measurement time was set to 2 s per stopper, and 1 s was required to switch to the next stopper. Therefore, the total analysis time for 100 stoppers was 300 s. Stoppers tainted by TCA levels differing by several orders of magnitude were processed.

**Figure 8:**
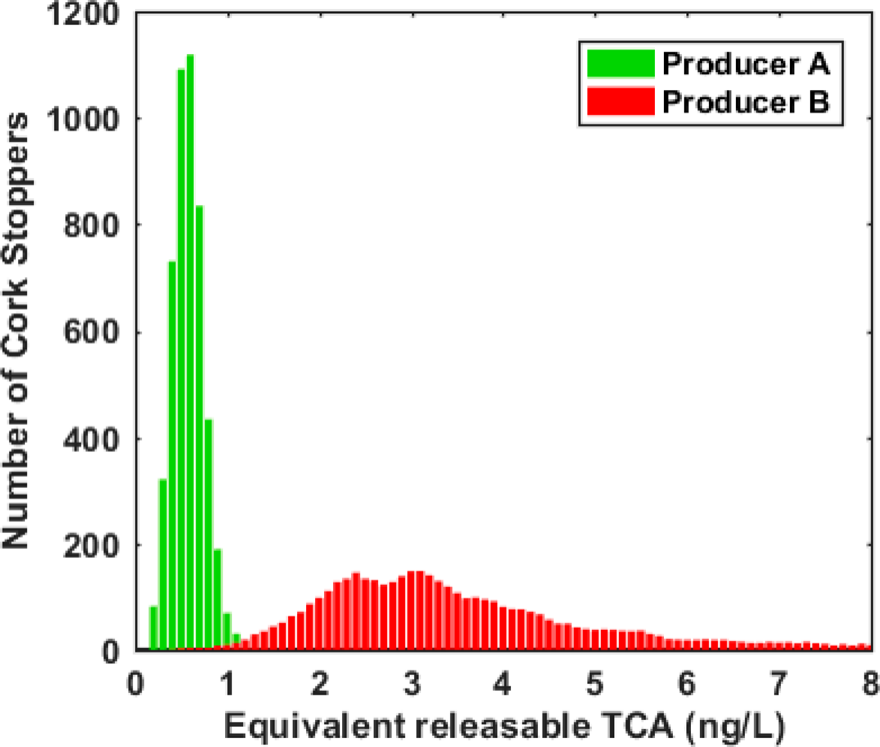
Example of TCA analysis results of two natural cork stopper batches (from two different producers) measured by VCA. Each batch consisted of 5000 natural cork stoppers having the same dimensions. The total analysis time for each batch has been 250 min. Histograms have been generated using bins of 0.1 ng/L.

Given the extremely short measurement time for single cork stoppers, three sets of natural cork stoppers from different producers were measured, for a total of 10100 natural cork stoppers. The results for the smallest set, consisting of 100 natural cork stoppers, are reported in Figure 7. The cork stoppers were measured sequentially one after the other, and remarkably, the total measurement time for the whole experiment was 5 min. This batch included several cork stoppers that were highly contaminated with TCA as well as cork stoppers having only a slight contamination, and some cork stoppers showed a TCA signal below the detection limit. Figure 8 summarizes the TCA quantification in two different batches, each consisting of 5000 cork stoppers. The total measurement time for each batch was 4 h and 10 min. For comparison, if the same experiment was carried out employing the ISO method, it would have required more than four months of constant analysis.

Moreover, the present method is nondestructive, and no visual damage or deformation appeared on the cork stoppers (Tables S2–S4 and Figure S2). Therefore, the samples can still be used (and, e.g., sold) after the analysis, provided a remoisturizing step is carried out in order to restore the moisture content. The latter procedure is very common within the cork industry. The histograms reported in Figure 8 indicate that the first batch is characterized by an average lower TCA content than the second batch. Over 99% of the cork stoppers were contaminated with <1 ng/L TCA and about 50% with <0.5 ng/L TCA, which is presently considered a limit for TCA-free corks within the cork industry.

On the contrary, the second batch had only a negligible percentage of TCA-free corks, while almost all of them were contaminated with >1 ng/L TCA.

The present technique can be useful in an industrial scenario to screen natural cork stoppers based on their TCA contamination level before they are sold or in a quality control laboratory in order to quickly assess the TCA incidence in cork batches.

## Conclusions

A novel nondestructive technique for quantification of the TCA contamination in single cork stoppers has been presented. The approach by far exceeds the performances of existing analytical methods in terms of speed while having comparable detection limits for TCA. It correlates with releasable TCA quantification using standard methods. The new technique possesses the potential to be a breakthrough for the cork and wine sector, providing the possibility of fast (3 s), single cork stopper preselection based on the rapid quantification of TCA contamination. Future developments include the investigation of other cork contaminants, which are simultaneously detected by the same technique.

## Supporting Information Available

Comparison of the Vocus spectrum of a TCA standard and TCA theoretical isotopic pattern. LOD, LOQ, and recovery. Pictures and physical and mechanical parameters of natural cork stoppers before and after measurement with a Vocus Cork Analyzer (PDF)
