# Supplementary material for: Thermal Desorption–Vocus Enables Online Nondestructive Quantification of 2,4,6-Trichloroanisole in Cork Stoppers below the Perception Threshold
Source: Anal Chem. 2020 Jun 10;92(14):9823–9. doi: 10.1021/acs.analchem.0c01326 (PMC8008374; doi:10.1021/acs.analchem.0c01326)
Supplement: Supplementary file 1 — ac0c01326_si_001.pdf [file ac0c01326_si_001.pdf]

Supporting Information

# Thermal Desorption - Vocus enables on-line non-destructive quantification of 2,4,6-trichloroanisole in cork stoppers below the perception threshold.

Luca Cappellin[*,‡,†,§], Felipe D. Lopez-Hilfiker[§], Veronika Pospisilova[§], Luigi Ciotti[§], Paolo Pastore[‡], Marc Gonin[§], Manuel A. Hutterli[§]

[‡]Dipartimento di Scienze Chimiche, Università degli Studi di Padova, via Marzolo 1, 35131 Padova, Italy
[†]Research and Innovation Centre, Fondazione Edmund Mach, via Mach 1, 38010 S. Michele a/A 38010, Italy
[§]Tofwerk AG, Schorenstrasse 39, CH-3645 Thun, Switzerland
*luca.cappellin@unipd.it

Table of content

Figure S1 .......... 2

Table S1 .......... 2

Table S2 .......... 2

Table S3 .......... 5

Table S4 .......... 6

Figure S2 .......... 7

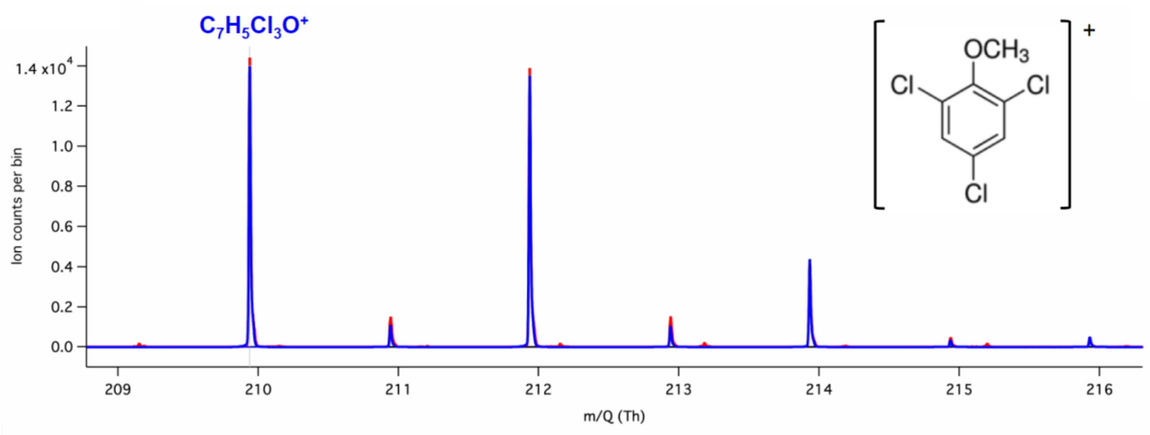


Figure S1. Vocus Cork Analyzer measured spectrum of TCA (red) and theoretical spectrum based on its isotopic distribution (blue).

Table S1. LOD, LOQ and Trueness of the present method. Absence of bias of the proposed method has been found (two tailed t-test, 95% confidence level).

| **LOD[a] (ng/L)** | **LOQ (ng/L)** | **Trueness[b]** |
|---|---|---|
| | | Recovery (% ± s[c]) |
| 0.05 | 0.15 | 102.7±3.2 |

[a] Estimated as described in the main text. [b] Estimated comparing Vocus Cork Analyzer with ISO for 171 natural cork stoppers.
[c] Estimated as standard uncertainty.

Table S2. Average diameter (n=2), length, weight, and specific weight before measurement with Vocus Cork Analyzer and after measurement and reconditioning (48h at (20 ± 1)°C and RH = 70%). Final relative humidity of corks 5±1%. Each row represents a different cork stopper. Cork stopper belonged to visual grade Extra, 1st, or 2nd. Measurements of physical parameters were performed using 150 Mitutoyo ABS 150 digital caliper, Sartorius 4202i - 1S precision balance, ELVAmac RAPID 54 CORKER, AQUA-BOY Wood hygrometer. The table shows that no changes in the measured physical parameters were found (two tailed t-test, not significant at 95% confidence level).

| BEFORE MEASUREMENT | | | | AFTER MEASUREMENT AND RECONDITIONING | | | |
|---|---|---|---|---|---|---|---|
| Average Diameter (mm) | Length (mm) | Weight (g) | Specific Weight (kg/m$^3$) | Average Diameter (mm) | Length (mm) | Weight (g) | Specific Weight (kg/m$^3$) |
| 24.25 | 48.73 | 3.45 | 153.35 | 24.29 | 48.74 | 3.47 | 153.70 |
| 24.13 | 49.19 | 3.51 | 156.10 | 24.17 | 49.20 | 3.53 | 156.44 |
| 23.80 | 49.37 | 3.45 | 157.14 | 23.84 | 49.38 | 3.45 | 156.52 |
| 24.19 | 49.24 | 3.55 | 156.87 | 24.26 | 49.24 | 3.54 | 155.59 |

| | | | | | | | |
|---|---|---|---|---|---|---|---|
| 24.30 | 48.74 | 3.11 | 137.64 | 24.32 | 48.73 | 3.10 | 136.95 |
| 23.95 | 49.37 | 3.48 | 156.46 | 23.94 | 49.37 | 3.51 | 158.01 |
| 23.91 | 48.96 | 4.26 | 193.87 | 23.93 | 48.95 | 4.24 | 192.59 |
| 24.25 | 49.32 | 3.98 | 174.79 | 24.25 | 49.33 | 4.00 | 175.64 |
| 23.72 | 48.72 | 3.76 | 174.65 | 23.79 | 48.72 | 3.78 | 174.62 |
| 23.80 | 49.23 | 4.05 | 185.00 | 23.76 | 49.24 | 4.03 | 184.59 |
| 23.74 | 49.07 | 3.14 | 144.63 | 23.70 | 49.09 | 3.17 | 146.44 |
| 24.14 | 49.13 | 3.54 | 157.50 | 24.08 | 49.11 | 3.54 | 158.35 |
| 23.80 | 49.40 | 3.78 | 172.00 | 23.78 | 49.39 | 3.76 | 171.41 |
| 23.79 | 49.26 | 3.79 | 173.16 | 23.80 | 49.25 | 3.80 | 173.51 |
| 24.04 | 48.74 | 3.69 | 166.79 | 24.07 | 48.74 | 3.67 | 165.55 |
| 23.82 | 49.36 | 3.24 | 147.30 | 23.80 | 49.34 | 3.22 | 146.76 |
| 23.97 | 49.21 | 4.09 | 184.26 | 23.97 | 49.21 | 4.11 | 185.16 |
| 23.89 | 49.38 | 4.22 | 190.65 | 23.95 | 49.37 | 4.23 | 190.26 |
| 24.20 | 49.07 | 3.69 | 163.56 | 24.24 | 49.06 | 3.72 | 164.31 |
| 23.90 | 48.67 | 3.80 | 174.03 | 23.89 | 48.69 | 3.77 | 172.73 |
| 23.77 | 49.28 | 4.07 | 186.19 | 23.73 | 49.27 | 4.05 | 185.86 |
| 23.79 | 49.04 | 3.74 | 171.57 | 23.80 | 49.06 | 3.75 | 171.81 |
| 24.03 | 48.86 | 3.53 | 159.30 | 24.05 | 48.85 | 3.52 | 158.69 |
| 24.15 | 49.37 | 3.27 | 144.66 | 24.11 | 49.37 | 3.25 | 144.19 |
| 23.76 | 48.89 | 3.55 | 163.84 | 23.74 | 48.89 | 3.55 | 164.04 |
| 24.11 | 48.69 | 3.42 | 153.92 | 24.11 | 48.69 | 3.41 | 153.40 |
| 24.17 | 49.14 | 3.48 | 154.35 | 24.13 | 49.13 | 3.48 | 154.96 |
| 24.05 | 49.32 | 3.44 | 153.60 | 24.05 | 49.31 | 3.46 | 154.46 |
| 24.15 | 48.68 | 3.58 | 160.55 | 24.18 | 48.69 | 3.59 | 160.63 |
| 24.26 | 49.12 | 3.94 | 173.60 | 24.24 | 49.13 | 3.95 | 174.22 |
| 23.76 | 49.10 | 3.34 | 153.42 | 23.76 | 49.11 | 3.31 | 152.01 |
| 24.10 | 48.97 | 3.34 | 149.52 | 24.12 | 48.98 | 3.36 | 150.20 |
| 24.29 | 49.06 | 3.15 | 138.56 | 24.27 | 49.08 | 3.15 | 138.79 |
| 24.31 | 49.31 | 4.13 | 180.52 | 24.27 | 49.29 | 4.13 | 181.12 |
| 23.98 | 48.76 | 4.26 | 193.45 | 24.00 | 48.78 | 4.24 | 192.14 |
| 24.14 | 49.02 | 3.61 | 160.90 | 24.05 | 49.00 | 3.59 | 161.28 |
| 24.28 | 48.92 | 3.10 | 136.92 | 24.22 | 48.93 | 3.10 | 137.57 |
| 23.86 | 48.63 | 3.14 | 144.41 | 23.87 | 48.63 | 3.12 | 143.37 |
| 24.11 | 48.80 | 4.02 | 180.44 | 24.14 | 48.80 | 4.05 | 181.41 |
| 23.80 | 49.20 | 4.19 | 191.51 | 23.82 | 49.19 | 4.19 | 191.23 |
| 23.90 | 48.80 | 3.41 | 155.82 | 23.90 | 48.79 | 3.41 | 155.79 |
| 24.26 | 48.76 | 3.25 | 144.25 | 24.29 | 48.77 | 3.25 | 143.87 |
| 24.04 | 49.26 | 4.38 | 195.98 | 24.03 | 49.26 | 4.36 | 195.24 |
| 23.75 | 49.34 | 3.85 | 176.13 | 23.75 | 49.34 | 3.82 | 174.84 |
| 23.93 | 48.71 | 3.36 | 153.37 | 23.95 | 48.73 | 3.33 | 151.75 |
| 23.73 | 48.71 | 3.45 | 160.15 | 23.70 | 48.71 | 3.43 | 159.69 |

| | | | | | | | |
|---|---|---|---|---|---|---|---|
| 23.80 | 48.77 | 3.99 | 183.90 | 23.78 | 48.78 | 3.96 | 182.86 |
| 24.15 | 49.11 | 3.10 | 137.86 | 24.11 | 49.10 | 3.09 | 137.90 |
| 24.00 | 48.84 | 4.31 | 195.15 | 23.99 | 48.85 | 4.34 | 196.55 |
| 24.03 | 49.11 | 3.21 | 144.18 | 24.01 | 49.13 | 3.23 | 145.27 |
| 23.83 | 49.14 | 3.41 | 155.59 | 23.80 | 49.16 | 3.43 | 156.83 |
| 24.12 | 49.14 | 4.03 | 179.48 | 24.15 | 49.15 | 4.03 | 179.08 |
| 24.24 | 49.01 | 4.19 | 185.33 | 24.25 | 49.01 | 4.17 | 184.30 |
| 23.89 | 48.97 | 4.03 | 183.67 | 23.92 | 48.96 | 4.03 | 183.17 |
| 23.77 | 48.79 | 4.22 | 194.99 | 23.74 | 48.79 | 4.20 | 194.48 |
| 23.97 | 48.87 | 3.45 | 156.51 | 24.02 | 48.87 | 3.44 | 155.34 |
| 24.09 | 48.94 | 4.35 | 195.01 | 24.12 | 48.96 | 4.35 | 194.53 |
| 24.20 | 48.65 | 3.73 | 166.76 | 24.23 | 48.66 | 3.75 | 167.13 |
| 24.01 | 48.76 | 4.38 | 198.40 | 24.00 | 48.76 | 4.41 | 199.92 |
| 23.98 | 48.61 | 3.38 | 153.96 | 24.00 | 48.62 | 3.40 | 154.64 |
| 24.26 | 49.14 | 3.76 | 165.60 | 24.26 | 49.15 | 3.77 | 166.01 |
| 24.29 | 48.79 | 4.06 | 179.65 | 24.29 | 48.81 | 4.05 | 179.13 |
| 24.13 | 49.13 | 3.15 | 140.26 | 24.10 | 49.12 | 3.13 | 139.75 |
| 24.20 | 48.80 | 4.20 | 187.19 | 24.18 | 48.79 | 4.18 | 186.57 |
| 24.13 | 48.95 | 3.16 | 141.22 | 24.15 | 48.96 | 3.18 | 141.80 |
| 24.30 | 48.95 | 4.32 | 190.37 | 24.27 | 48.94 | 4.31 | 190.36 |
| 24.16 | 48.65 | 3.59 | 161.03 | 24.16 | 48.65 | 3.60 | 161.48 |
| 23.79 | 49.05 | 4.20 | 192.71 | 23.77 | 49.07 | 4.19 | 192.50 |
| 23.68 | 48.80 | 3.92 | 182.40 | 23.67 | 48.79 | 3.90 | 181.73 |
| 23.93 | 49.25 | 4.25 | 191.95 | 23.94 | 49.24 | 4.23 | 190.93 |
| 24.05 | 48.96 | 4.03 | 181.27 | 24.09 | 48.97 | 4.03 | 180.63 |
| 24.29 | 49.03 | 3.52 | 154.93 | 24.28 | 49.04 | 3.50 | 154.21 |
| 23.70 | 49.16 | 3.78 | 174.37 | 23.74 | 49.18 | 3.79 | 174.17 |
| 24.27 | 49.34 | 4.28 | 187.58 | 24.31 | 49.32 | 4.25 | 185.73 |
| 24.23 | 48.64 | 3.52 | 156.95 | 24.26 | 48.64 | 3.51 | 156.11 |
| 24.14 | 48.60 | 3.47 | 156.00 | 24.13 | 48.61 | 3.44 | 154.81 |
| 23.79 | 49.39 | 3.55 | 161.70 | 23.79 | 49.38 | 3.57 | 162.64 |
| 23.77 | 49.38 | 3.55 | 162.01 | 23.82 | 49.36 | 3.52 | 160.03 |
| 23.90 | 49.12 | 4.23 | 192.03 | 23.93 | 49.12 | 4.21 | 190.65 |
| 23.90 | 49.29 | 3.45 | 156.02 | 23.91 | 49.27 | 3.47 | 156.85 |
| 23.83 | 49.22 | 3.45 | 157.16 | 23.84 | 49.23 | 3.47 | 157.91 |
| 24.16 | 48.81 | 3.43 | 153.29 | 24.15 | 48.81 | 3.43 | 153.41 |
| 23.98 | 48.99 | 3.20 | 144.63 | 24.02 | 48.98 | 3.20 | 144.24 |
| 23.86 | 48.72 | 3.80 | 174.44 | 23.84 | 48.71 | 3.78 | 173.85 |
| 23.88 | 49.16 | 3.67 | 166.75 | 23.89 | 49.17 | 3.67 | 166.58 |
| 24.05 | 48.87 | 4.37 | 196.84 | 24.07 | 48.86 | 4.37 | 196.56 |
| 23.75 | 48.62 | 3.53 | 163.96 | 23.75 | 48.61 | 3.56 | 165.31 |
| 24.03 | 48.85 | 4.26 | 192.29 | 24.04 | 48.85 | 4.25 | 191.68 |

| | | | | | | | |
|---|---|---|---|---|---|---|---|
| 24.16 | 49.03 | 3.40 | 151.26 | 24.17 | 49.03 | 3.38 | 150.31 |
| 23.68 | 48.82 | 3.27 | 152.09 | 23.74 | 48.80 | 3.24 | 150.06 |
| 23.86 | 48.64 | 3.55 | 163.30 | 23.89 | 48.66 | 3.53 | 161.91 |
| 24.18 | 49.06 | 3.37 | 149.59 | 24.15 | 49.05 | 3.35 | 149.10 |
| 23.85 | 48.71 | 3.11 | 142.91 | 23.82 | 48.69 | 3.09 | 142.41 |
| 24.07 | 48.78 | 3.71 | 167.21 | 24.13 | 48.77 | 3.73 | 167.31 |
| 24.33 | 48.95 | 4.32 | 189.90 | 24.27 | 48.94 | 4.35 | 192.21 |
| 24.12 | 49.31 | 3.66 | 162.44 | 24.16 | 49.30 | 3.68 | 162.89 |
| 23.75 | 48.93 | 3.54 | 163.31 | 23.76 | 48.94 | 3.53 | 162.68 |
| 24.02 | 49.35 | 3.91 | 174.92 | 23.97 | 49.37 | 3.92 | 176.03 |
| 23.77 | 49.10 | 3.43 | 157.42 | 23.69 | 49.11 | 3.45 | 159.38 |
| 24.28 | 49.29 | 3.64 | 159.56 | 24.23 | 49.28 | 3.64 | 160.26 |

Table S3. 24x49 mm cork stoppers not measured with Vocus Cork Analyzer. Determination of dimensional recovery after compression according to ISO 9727-4:2007. Cylindrical cork stoppers - Physical tests - Part.4. ISO 16420:2013. (Cork stoppers for still wines. Mechanical and physical specifications.) prescribes a dimensional recovery: > 90% after 3 min following compression to 16 mm.

| Sample | Average Diameter before compression (mm) | Length (mm) | Weight (g) | Specific weight (kg/m$^3$) | Average Diameter 3 min after compression (mm) | RECOVERY % | DENSITY CLASS | AVERAGE RECOVERY % |
|---|---|---|---|---|---|---|---|---|
| 1A | 23.94 | 48.66 | 3.13 | 143.04 | 23.45 | 97.95 | A | 98,06 |
| 2A | 24.05 | 48.90 | 3.25 | 146.36 | 23.57 | 98.00 | A | |
| 3A | 23.82 | 48.64 | 3.14 | 144.69 | 23.38 | 98.15 | A | |
| 4A | 24.11 | 48.96 | 3.15 | 140.89 | 23.65 | 98.11 | A | |
| 5A | 24.01 | 48.78 | 3.34 | 151.23 | 23.50 | 97.86 | B | 97,58 |
| 6A | 24.08 | 49.26 | 3.27 | 145.59 | 23.48 | 97.51 | B | |
| 7A | 24.16 | 48.77 | 3.36 | 150.48 | 23.54 | 97.45 | B | |
| 8A | 23.96 | 48.65 | 3.37 | 153.74 | 23.36 | 97.50 | B | |
| 9A | 24.26 | 49.26 | 3.54 | 155.33 | 23.46 | 96.70 | C | 96,99 |
| 10A | 23.83 | 49.12 | 3.38 | 154.53 | 23.14 | 97.10 | C | |
| 11A | 24.01 | 49.04 | 3.45 | 155.56 | 23.33 | 97.15 | C | |
| 12A | 23.85 | 49.12 | 3.39 | 154.64 | 23.13 | 97.00 | C | |
| 13A | 24.06 | 49.37 | 3.74 | 166.78 | 23.25 | 96.65 | D | 96,76 |
| 14A | 23.74 | 49.14 | 3.77 | 173.41 | 22.98 | 96.80 | D | |
| 15A | 23.79 | 48.78 | 3.83 | 176.54 | 23.10 | 97.10 | D | |
| 16A | 24.19 | 48.66 | 3.60 | 161.13 | 23.34 | 96.49 | D | |
| 17A | 23.91 | 49.38 | 4.11 | 185.55 | 23.10 | 96.59 | E | 96,47 |
| 18A | 24.23 | 49.21 | 4.37 | 192.54 | 23.38 | 96.49 | E | |
| 19A | 23.87 | 49.29 | 4.03 | 182.92 | 23.06 | 96.61 | E | |
| 20A | 23.92 | 48.73 | 4.09 | 186.64 | 23.01 | 96.20 | E | |

Table S4. 24x49 mm cork stoppers after measurement with Vocus Cork Analyzer and reconditioning (48h at (20 ± 1)°C and RH = 70%). Determination of dimensional recovery after compression according to ISO 9727-4:2007. Cylindrical cork stoppers - Physical tests - Part.4. ISO 16420:2013. (Cork stoppers for still wines. Mechanical and physical specifications.) prescribes a dimensional recovery: > 90% after 3 min following compression to 16 mm. Comparison of Tables S2 and S3 shows that no differences in recovery were caused by the Vocus Cork Analyzer (two tailed t-test, not significant at 95% confidence level).

| Sample | Average Diameter before compression (mm) | Length (mm) | Weight (g) | Specific weight (kg/m$^3$) | Average Diameter 3 min after compression (mm) | RECOVERY % | DENSITY CLASS | AVERAGE RECOVERY % |
|---|---|---|---|---|---|---|---|---|
| 1B | 23.90 | 49.10 | 3.24 | 147.27 | 23.41 | 97.95 | A | 98.16 |
| 2B | 24.20 | 49.01 | 3.14 | 139.44 | 23.76 | 98.20 | A | |
| 3B | 24.26 | 49.20 | 3.22 | 141.63 | 23.82 | 98.19 | A | |
| 4B | 23.85 | 49.13 | 3.19 | 145.49 | 23.44 | 98.30 | A | |
| 5B | 24.16 | 49.01 | 3.26 | 145.18 | 23.60 | 97.66 | B | 97.61 |
| 6B | 23.79 | 49.08 | 3.40 | 155.87 | 23.24 | 97.69 | B | |
| 7B | 23.80 | 48.87 | 3.21 | 147.57 | 23.21 | 97.54 | B | |
| 8B | 23.94 | 48.60 | 3.34 | 152.45 | 23.36 | 97.56 | B | |
| 9B | 23.80 | 48.93 | 3.45 | 158.60 | 23.10 | 97.06 | C | 97.05 |
| 10B | 23.79 | 49.36 | 3.48 | 158.47 | 23.02 | 96.76 | C | |
| 11B | 23.75 | 48.67 | 3.42 | 158.57 | 23.08 | 97.16 | C | |
| 12B | 23.77 | 48.96 | 3.48 | 160.36 | 23.11 | 97.20 | C | |
| 13B | 23.84 | 49.01 | 3.76 | 172.13 | 23.06 | 96.75 | D | 96.67 |
| 14B | 24.00 | 49.38 | 3.74 | 167.51 | 23.18 | 96.58 | D | |
| 15B | 23.92 | 48.91 | 3.83 | 174.33 | 23.17 | 96.86 | D | |
| 16B | 23.82 | 48.89 | 3.76 | 172.47 | 22.98 | 96.49 | D | |
| 17B | 24.03 | 48.86 | 4.32 | 194.99 | 23.14 | 96.30 | E | 96.43 |
| 18B | 23.95 | 48.82 | 4.03 | 183.33 | 23.14 | 96.62 | E | |
| 19B | 24.10 | 49.13 | 4.16 | 185.71 | 23.27 | 96.56 | E | |
| 20B | 23.79 | 48.72 | 4.25 | 196.10 | 22.89 | 96.24 | E | |

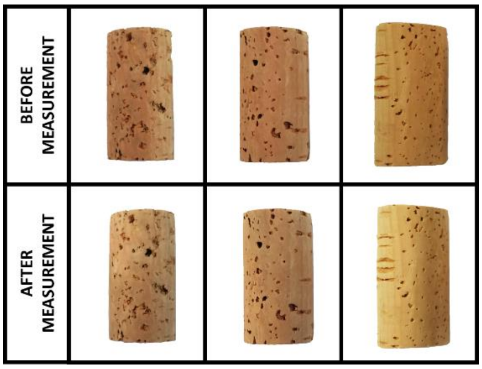


Figure S2. Examples of pictures of cork stoppers before and after the measurements with Vocus Cork Analyzer. No visual differences were found. Apparent distortions or color changes are due to light, camera angles, and other picture defects.
